# Verifications

**Published:** 1890-10

**Authors:** Wm. Jefferson Guernsey

**Affiliations:** Philadelphia


### VERIFICATIONS.

WM. JEFFERSON GUERNSEY, M. D., PHILADELPHIA.

HYDROPHOBIA.—Diseases that rarely tend toward spontaneous resolution, and in the treatment of which allopathy is acknowledged by its adherents to be of doubtful efficacy are daily cured by Homœopathy, and it is not only gratifying, but exceedingly beneficial to one's faith in the only *law* of cure to read of such cases as that reported by Dr. W. A. Yingling, page 400, in THE HOMŒOPATHIC PHYSICIAN of September.

Another such once made a vivid impression upon me. A boy of about nine years while teasing a "pet" rat was bitten by the "varmint" on the first finger of the left hand. I am sorry that my record does not state how long a time had elapsed before I was called in, but I found him, as I saw at once, horribly ill. Is it not the experience of all physicians that the first glance at some cases fills him with awe? I *felt* that I had an undesirable case in hand, and one that had not been too early placed under treatment. The boy could talk only with great difficulty; his teeth were not quite firmly locked, but he was conscious of an almost momentary aggravation of the difficulty in separating the jaws; his neck was so stiff as to render any motion of the head almost impossible. With this was a nervous excitement and marked tremor of the body. I preferred *Hypericum* to *Ledum,* on account of there being more tenderness about the wound than its appearance would indicate, and gave him the 500th of Tafel's in water every fifteen minutes. This was about eight P. M., and his mother stated the following morning that he had been very feverish and restless till three o'clock, when, as he "looked" better and was sleepy, she did not give the medicine but about every two hours, when he moved or awoke. I found him sitting up in bed at nine o'clock amusing himself and his mother by opening and shutting his mouth and turning his head about, and quite indignant because he could not be up and dressed, which was allowed on the day following.

PAIN IN THE SHIN BONES.—Now, while in the line of verifications, may I be permitted to refer to page 421 of the same number? Dr. S. Mills Fowler thereon states as follows: "* * * terrible pains in the shin bones. * * * Hering gives as a characteristic of Lachesis, 'much pain of an aching kind in the shin bones only.' I have, three different times since the above observation, verified this symptom clinically, and have come to regard it as 'the red string' for Lachesis." Now, if you have Vol. VII of THE HOMŒOPATHIC PHYSICIAN before you, please turn to page 62 and read, in an article by myself a comment on this. But I see you have not, so I will copy it here: "Whenever I find a 'throat case' having these pains I say confidently to myself, either the left side of the throat is worse or the trouble has commenced there, and have *never failed to find it so*. Further than this, I have numbers of times been called to see a person suffering from and complaining mainly of those pains with fever (which had been preceded by a chill), and with slight huskiness or 'thickness' of the voice, who would not complain at all of the throat, or perhaps even assert that there was little or no trouble there. Looking, I would find marked ulceration or deposit, and always worse on the left side." I sincerely trust that this will not come to the eye of an allopath, for I can imagine him asking what possible relation the pains in the shin bones have to the left side of the throat, and I could not, I am sure, tell him. But just here, where allopathy is a blank, Homœopathy places *Lachesis*, and the circuit is complete.

